# Attentional bias for negative, positive, and threat words in current and remitted depression

**DOI:** 10.1371/journal.pone.0205154

**Published:** 2018-10-31

**Authors:** Hermien J. Elgersma, Ernst H. W. Koster, Lonneke A. van Tuijl, A. Hoekzema, Brenda W. J. H. Penninx, Claudi L. H. Bockting, Peter J. de Jong

**Affiliations:** 1 Department of Clinical Psychology & Experimental Psychopathology, University of Groningen, Groningen, the Netherlands; 2 Department of Experimental-Clinical and Health Psychology, Ghent University, Ghent, Belgium; 3 Institute of Psychiatry, Psychology and Neuroscience, King’s College London, London, United Kingdom; 4 Department of Research Support, Faculty of Behavioral and Social Sciences, University of Groningen, Groningen, The Netherlands; 5 Department of Psychiatry/EMGO Institute for Health and Care Research, VU University Medical Centre, Amsterdam, the Netherlands; 6 Department of Psychiatry, Academic Medical Center, University of Amsterdam, Amsterdam, the Netherlands; Leiden University, NETHERLANDS

## Abstract

**Background:**

The aim of this study was to improve our understanding of the underlying mechanisms in the maintenance of depression. We examined attentional bias (AB) for negative and positive adjectives and general threat words in strictly-defined clinical groups of participants with pure Major Depressive Disorder (MDD) without a history of anxiety disorders (AD), mixed MDD and AD, and remitted participants.

**Method:**

We investigated both stimulus specificity and time course of AB in these groups, adopting a cross-sectional design. Data were drawn from the large scale Netherlands Study of Depression and Anxiety (NESDA), from which we selected all participants with pure current MDD without a history of AD (n = 29), all participants with current MDD and co-morbid AD(s) (n = 86), all remitted MDD participants (n = 294), and a comparison group without (a history of) MDD or ADs (n = 474). AB was measured with an Exogenous Cueing Task covering short and long presentation times (500 and 1250 ms) and 4 stimulus types (negative, positive, threat, neutral).

**Results:**

Both traditional and trial level (dynamic) AB scores failed to show an AB for negative adjectives in participants with MDD or mixed MDD/AD. Specifically for long duration trials (1250 ms), remitted participants showed a larger AB traditional score (albeit the actual score still being negative) than the comparison group. The mixed MDD/AD group showed a higher trial-level AB score away from positive adjectives (1250 ms) than the comparisons. In addition, the mixed MDD/AD group showed higher and more variable trial-level AB scores away from short and towards longer presented general threat words together with a non-significant tendency to show less negative traditional AB scores for threat trials (500 ms) than the comparison group.

**Conclusions:**

All in all, the findings do not corroborate the view that an AB towards negative or away from positive adjectives is critically involved in currently depressed individuals. Yet, the relatively high (less negative) AB score for negative adjectives in remitted individuals points to the possibility that an AB for negative information may be involved as a risk factor in the recurrence of MDD.

## Introduction

Depression is a common and severe mental disorder. Despite immense research investment to improve existing treatment and prevention interventions, meta-analyses show that effect-sizes of these treatments remain rather small [1, 2, 3, 4]. Even if there is an initial improvement in response to treatment, this is often followed by relapse, with an increasing risk of relapse after every depressive episode [5]. Because of the recurrent nature of depression and its severe consequences, it is of great importance to improve our understanding of the mechanisms involved in the development, maintenance, and recurrence of depression.

Cognitive theories emphasize the role biased processing of affective information has in the development and maintenance of depression [6, 7, 8, 9, 10]. Many of these cognitive models include attentional bias (AB) as both a contributing and a maintaining factor to depression (e.g.,[11]. Attentional bias is described as the preferential attention toward certain types of (emotional) information in the environment. Consistent with the view that AB is involved in depression, findings of a meta-analysis covering 29 empirical studies using emotional Stroop or dot probe tasks in individuals with depressive complaints (clinical depression, nonclinical dysphoria, induced depressive mood) havorted the view that depression is associated with biased attention to negative information [12]. Because many patients with MDD have comorbid anxiety disorders, it is important to verify whether AB can indeed be attributed to MDD per se. In support of the view that AB in MDD is not merely due to comorbid anxiety disorders, one of the studies that was included in this meta-analysis showed that MDD participants with no comorbid panic disorder or social phobia (n = 88) displayed a stronger AB for sad faces (within the context of a visual probe study with happy, sad, and angry faces) than participants with only social phobia (n = 35) and participants without MDD or social phobia (n = 55) [13]. There is also evidence that depression is characterized by a lack of attention towards positive information. For instance, in the context of a free-viewing task, clinically depressed young adults spent less time looking at positive images than never depressed participants [14]. A similar pattern has been found in dysphoric individuals (sub-clinically depressed) [15, 16]. Interestingly, there is also some evidence of a predictive relationship in participants with major depressive disorder (MDD): A difficulty in disengaging attention from sad faces has been related to sustained negative mood, as measured in an eye-tracking study [17]. Furthermore, in participants with MDD, higher levels of AB for sad faces, as measured with an exogenous cueing task (ECT), was related to impaired mood recovery in reaction to a sad mood induction [18].

Taken together, the available evidence suggests that depressed individuals are characterized by heightened AB for negative information and lowered AB for positive information. However, since most evidence is based on analogue research or small heterogeneous clinical samples (e.g., [19], it remains important to test the robustness of this pattern in well-defined clinical samples. In addition, there are several important questions regarding AB that remain. Addressing these issues is the main aim of this study as described next in more detail.

The first aim of this study is to examine AB and its stimulus specificity within an adult sample of clinically diagnosed patients with MDD. Thus far, AB research in depression has mainly focused on the attentional preference for negative versus positive information (for a review, see [11], whereas studies investigating AB in anxiety disorders (AD)s typically relied on (disorder-specific) threatening information (e.g., [20, 21,22]. Only a few studies have looked at AB for threatening information in MDD. One of these studies presented participants with sets of depression-related, anxiety-related, positive, and neutral images and tracked participants’ eye-movements. Previously depressed individuals and dysphoric individuals spent less time looking at positive images than never-depressed persons. Importantly, previously depressed individuals spent more time looking at anxiety-relevant images during a free-viewing task (such as scenes of people being threatened with weapons, people with physical injuries, dangerous situations) than never depressed individuals [23]. In addition, an earlier study using a visual probe task depicting words, found that participants who were diagnosed with Dysthymia or MDD showed an AB for socially threatening words [19]. Together, these findings point to the relevance of using different categories of emotionally relevant stimuli to examine AB in depression. The process of AB might not only be specific for diagnoses but could also play a transdiagnostic role in psychopathology. Therefore, the current study included both depression-relevant and threat-related stimuli to test whether participants with a clinically-diagnosed MDD show an AB not only for depression-related stimuli but also for threat-related stimuli. In addition, we included positive stimuli to test whether AB in MDD is not only characterized by enhanced attention for negative information but also an attenuated bias for positive information.

The second aim of this study relates to the temporal unfolding of attentional bias as stimuli are presented for longer presentation times. Earlier research provided evidence indicating that it may be critical to take the time course of AB into consideration when examining the relevance of AB in the context of depression. A study testing AB in dysphoric vs. non-dysphoric students showed that the AB for negative adjectives was especially prominent during relatively long presentation times (1500 ms), and absent during relatively short presentation times (250 ms) [24]. Subsequent research among nonclinical participants found similar results [25] suggesting that AB in depression may reflect a difficulty in disengaging from negative information rather than enhancing orientation/engagement. We therefore measured AB both for shorter and longer presentation times. In addition, we tested whether the temporal unfolding of AB in MDD differs across stimulus type [22].

The third aim of this study relates to the observation that MDD and AD often co-occur[26]. Given that depressed patients tend to also score high on anxiety questionnaires, it can often not be ruled out that any observed AB is mainly driven by anxiety rather than depression levels. Yet, a recent analogue study showed that participants who reported both symptoms of anxiety and depression displayed an AB for emotional words, whereas participants who only reported heightened symptoms of depression did not [27]. To improve our understanding of how AB may contribute to depression it would, therefore, be important to differentiate between individuals with and without comorbid AD (cf. [13]. Therefore, the current study distinguished between groups of participants with MDD (and no AD) and participants with MDD and comorbid AD. In this way we were able to examine whether the pattern of AB in pure MDD participants differed from that in individuals with comorbid AD.

The fourth aim of this study was to investigate AB in remitted depressed (rMDD) participants. It has been hypothesized that rMDD individuals may still be characterized by an AB towards negative and away from positive stimuli. This prediction is based on the hypothesis that AB is a stable vulnerability factor that renders individuals vulnerable for the development of depression. Therefore, this vulnerability factor will still be present after recovery of depression thereby contributing to the development of recurrent depressive episodes [8, 11, 28]. In line with this view, both currently depressed and rMDD individuals selectively attended to sad faces when measured with a dot-probe task [29], whereas healthy comparisons avoided sad faces and oriented toward the happy faces. In a similar vein, both dysphoric and rMDD individuals spent significantly less time attending to positive images (such as scenes of people smiling, or kittens) and more to depression-images (e.g., scenes of people appearing sad, unhappy, or images of neglected animals, etc.) than never depressed individuals [23]. rMDD individuals also attended to anxiety-related images (e.g., scenes of threat and injury, people being threatened by weapons) more than never depressed individuals. These studies seem to support the hypothesis that even after recovery of depression, heightened AB for negative and lowered AB for positive information remains, possibly increasing the risk of relapse or recurrence. In this study, we tested the robustness of these earlier findings by comparing a large group of rMDD to never-depressed individuals with regard to their AB for positive and negative adjectives, as well as for general threat words.

Given recent findings highlighting the potential relevance of fluctuations in AB[30], in the current study we not only relied on traditional AB indices, but also took the trial-by-trial AB variation into account. Zvielli et al. [30]proposed that the concept of AB as a stable process (based on an averaged AB across trials within a task) may not reflect the dynamic expression of AB; “AB may be expressed in fluctuating, phasic bursts, toward or away from target stimuli over time” (p.774). Zvielli et al. [30]argued that previous findings concerning AB were explained by a failure to take the importance of the dynamic nature of AB into account. Building further on previous concepts and measurements of AB, these authors proposed trial-level bias scores (TL-BS) representing variance in AB towards the target stimuli, variance in AB away from the target stimuli, maximum AB towards the target stimuli, maximum AB away from the target stimuli, and overall variability in AB towards and away from the target stimuli. It seems plausible that the mixed results of AB studies in participants with MDD, rMDD and/or the mixed participants can be untangled using the temporal dynamics of AB. Based on this view, Zvielli, et al. [31] recently reanalyzed data of a published dot probe study with sad, positive, and neutral faces that showed no differential effects on the basis of traditional AB indices[32]. Interestingly, the results of this re-analysis showed that rMDD participants were characterized by higher levels of TL-BS, specifically increased variability, than non-depressed individuals. This may reflect a greater dysregulation of attentional processing of emotional information in rMDD individuals [31]. Although the TL-BS approach showed promising results in terms of prognostic value, it also gave rise to major conceptual criticisms. A Monte Carlo simulation study demonstrated that TL-BS indices could be prone to result in false positive group differences; the differences between groups might in fact reflect differences in mean RT and or differences in overall SD [33]. Thus, any differential effect that may be found in the current study should be considered in light of these conceptual criticisms.

In sum, this study employed both traditional and recently proposed dynamic indices of AB to examine (i) the stimulus specificity and (ii) temporal unfolding of AB in MDD, (iii) whether the pattern of AB varies between MDD participants with and without a comorbid AD, (iv) whether AB is still present in participants who are recovered from MDD. We hypothesized that especially for the longer presentation times participants with MDD with and without AD’s would be characterized by stronger AB for negative adjectives than the comparison group. In addition, we hypothesized that the group of remitted participants would still have an AB, but less than the clinical groups (participants with MDD with and without ADs). We assumed that both patterns would especially be reflected on longer presentation times. We presumed that participants with MDD (with and without ADs) would show weaker AB for positive adjectives independent of presentation time. Finally, we hypothesized that the group of participants with co-morbid AD would be characterized by an AB for general threat words, especially when presented for a short duration.

## Materials and methods

This study was conducted as part of the Netherlands Study of Depression and Anxiety (NESDA) [34], an ongoing multi-centre, longitudinal cohort study designed to examine the long-term course and consequences of anxiety and depressive disorders. The study protocol was approved centrally by the Ethical Review Board of the VU University Medical Centre (protocol number 2013/183) and subsequently by local review boards of each participating centre (IRBs of the VU University Medical Center, the University Medical Center Groningen and the Leiden University Medical Center). After full verbal and written information about the study, written informed consent was obtained from all participants at the start of baseline assessment. Participants received written study information at home to read before they were invited to the face-to-face interview. Then during the face-to-face contact the written information was discussed and it was checked whether the information was completely understood. After this process, participants were asked to sign the consent form.

Baseline assessments started in September 2004. This study used data from the baseline and 2-year follow-up assessment (for details see [35]and the website www.nesda.nl) as the latter was the wave in which the ECT assessment was incorporated. The 2-year follow-up assessment included a wide range of outcome measures. Next to the ECT there were two other cognitive performance measures: A computerized working memory task (N-back) and an Implicit Association Task assessing self-anxious and self-depressed associations (for details see [35]and the website www.nesda.nl).

### Participants

Participants were recruited from the general population, through general practitioners, and in mental health care institutions, and included: healthy individuals with no history of psychiatric disorders, individuals at risk because of prior episodes, sub-threshold symptoms or family history, and individuals with a current first or recurrent MDD or AD. ADs were generalized anxiety disorder, panic disorder, social phobia, and agoraphobia.

General exclusion criteria were presence of a psychiatric disorder other than depressive or AD (e.g., psychosis, bipolar disorder, severe addictive disorder) or lack of fluency in Dutch. The current study concerns secondary analyses and the number of participants available for the current analyses was thus not based on the initial power analysis that determined the sample size of the cohort that was included in NESDA. Of the 2981 participants who were included at baseline, 2596 respondents participated in the 2-year follow-up measurements. The ECT was introduced during the 2-year follow up measurements and was completed by 2128 out of 2596 (81.97%) participants (61.9% female; mean age 43.63 years, SD = 14.06); 468 (18.02%) participants had no or too little ECT data (e.g., those interviewed over the phone or at home). Of the 2128 participants, we selected four subgroups: Group 1 (MDD) consisted of participants diagnosed with a current (in the last month) MDD, but without dysthymia and without a current AD or history of ADs (n = 29; 1.36%); Group 2 (mixed MDD/AD) consisted of participants diagnosed with a current (in the last month) major depressive disorder and a current anxiety disorder, but without dysthymia (n = 86; 4.04%); Group 3 (rMDD) consisted of participants with a history of MDD, but no current MDD nor dysthymia (in the last six months) and no current or history of anxiety disorders (n = 294; 13.81%); Group 4 (comparisons) consisted of healthy comparisons without a lifetime history of either anxiety or depressive disorders (n = 474; 22.27%) [35]. Part of the participants used medication such as antidepressants (AD). At baseline, a total of 748 (25.1% of the total sample) respondents were using antidepressants [34]. Because the NESDA is a naturalistic study, medication use was not under experimental control and analyses were conducted regardless of the use of medication. Although there is evidence that attentional biases can be responsive to medications in nonclinical populations (e.g., [36], it has been argued that within the context of clinical populations differences in AB are due to improved clinical status rather than to a medical treatment effect per se [37].

### Measures

#### Diagnostic assessment and other measures

The lifetime Composite International Diagnostic Interview (CIDI, lifetime version 2.1;[38]) was used to diagnose anxiety (panic disorder with agoraphobia, panic disorder without agoraphobia, agoraphobia without panic disorder, social phobia, generalized anxiety disorder) and depressive disorders according to DSM-IV criteria [39]. A disorder was considered current if participants suffered from it in the past month. Depressive symptoms were assessed using the 30-item Inventory of Depressive Symptoms Self-Report version (IDS-SR) [40]. The total score of the IDS-SR was used as an index for the severity of depression.

#### Exogenous cueing task (ECT)

The ECT is a reaction-time based attention task which was programmed using the E-Prime 1.0.2 software (Psychology Software Tools, Pittsburgh, PA). In the original exogenous cueing paradigm [41], participants are asked to detect a visual target presented at a left or right peripheral location. If a stimulus (a “cue”) precedes the target at the same spatial location, it is called a “valid” trial. On the remaining trials, the preceding stimulus is presented at the opposite spatial location of the target and thus invalidly cues the target’s location (“invalid” trials). In the emotional modification of this paradigm, the emotional value of the cue varies (i.e., emotional vs. neutral) which allows investigation of AB for an emotional cue.

The task used in this study was modelled after the ECT used in previous research on anxiety and depression (e.g., [42, 43, 44]). Stimuli were presented on a black background. During each trial, a white fixation cross was presented in the center of the screen. A white rectangle placeholder was presented (4 cm high x 10.5 cm long), both on the left and the right side of this fixation cross. The centers of these placeholders were located at 7.9 cm from the fixation cross. Cues (words) and targets (black squares) were presented in the center of the placeholders. Cues were 16 generally threatening words, 16 neutral words, 16 negative adjectives, and 16 positive adjectives (see Table 1). The threatening and neutral words were selected from earlier studies on AB [45, 46]. The negative and positive adjectives were selected from trait self-descriptors of depressive and manic persons, which were used in a study on AB in depression [47]. These words scored high on subjective familiarity in an earlier study investigating 740 Dutch words on affective and subjective familiarity [48]. See S1 Appendix for the stimulus words per stimulus type.

**Table 1:** Descriptives of participants per group; means and standard deviations.

GroupMeasures	Comparison (n = 474)	MDD (n = 29)	Mixed MDD/AD (n = 86)	rMDD(n = 294)
Age in years	43.77 (14.73)	43.76 (14.77)	43.34 (11.88)	44.50 (13.29)
Gender% female	59.5	65.5	67.3	64.6
IDS total score	6.24 (5.42)	23.71 (9.62)	32.00 (10.38)	10.98 (7.58)

Note:

Note that the numbers of the descriptives of the total groups and of the numbers in the final analyses differ slightly because of missing data (see data reduction). We used list wise deletion of missing data in the analyses.

IDS = Inventory of Depressive Symptoms-SR. For some of the participants IDS scores were missing. The number of participants with IDS total scores were as follows: Comparison (n = 470), MDD (n = 28, Mixed MDD/AD (n = 82), rMDD (n = 291).

MDD = group of participants with Major Depressive Disorder

Mixed MDD/AD = group of participants with Major Depressive Disorder and Anxiety Disorder

rMDD = group of participants who are remitted from Major Depressive Disorder

Each trial started with the presentation of the fixation cross and the two placeholders for 500 ms. Next, a word cue was presented in the left or right placeholder, for 500 ms (short presentation time) or 1250 ms (long presentation time). The target was presented until a response was made. Directly after responding, the next trial started. If a participant did not respond within 2 s, the next trial started. Participants were asked to focus their attention on the fixation cross and to respond as quickly and correctly as possible by pressing the left key of a response box when the target was presented on the left side or by pressing the right key of the response box when the target was presented on the right side. They were asked to ignore any other information that would be presented. To ensure that attention was indeed directed at the fixation cross, 20 digit trials were added, 10 in the first half of the task and 10 in the second half. In these trials, instead of a word cue, a digit appeared for 100 ms at the location of the fixation cross. Participants were instructed to press both the left and right key of the response box simultaneously upon appearance of a digit during these trials. When a participant gave a wrong response, a red rectangle with the word wrong in capitals appeared for 500 ms in the middle of the screen. If a participant gave the wrong number, also a rectangle with the words “missed digit!” in capitals appeared in the middle of the screen.

The instructions were presented on the computer and the task started with 10 practice trials. Participants were then given the opportunity to ask a research assistant questions before the actual task started. In the first half of the task, word cues were presented for 500 ms, in the second half of the task for 1250 ms. We preferred a fixed order to minimize method variance which we considered important in light of the prospective design of the NESDA study (cf.[49]). We started with the short duration trials as we anticipated that prior exposure to long duration word presentations could have a larger impact on performance during short duration trials than vice versa. Cues were presented at random on the right or left side of the fixation cross and every word cue was presented twice in each half of the task: Once in a valid trial (i.e., word cue is valid predictor for the target location), and once in an invalid trial. In total, the task consisted of 4 stimulus types x 16 exemplars x 2 valid/invalid x 2 presentation times = 256 word trials, 10 practice trials and 20 digit trials. The same fixed random order of trials was used for all participants to make the design more sensitive to individual differences. See S2 Appendix for an illustration of a valid and invalid trial of the ECT.

### Procedure

The assessments at baseline and follow-up were largely similar; they lasted between 3 and 5 hours and were conducted on one day. The two-year follow-up assessment consisted of a face-to-face clinic visit, in which baseline assessments–except those concerning stable concepts–were repeated. A few additional assessments, e.g. the ECT used in this study, were included. These other measurements are beyond the scope of this study (see [34, 35]for a detailed description). The assessments started with the CIDI-interview. After that, the ECT and questionnaires were completed. After completing the assessment, participants were compensated with a €15 gift certificate and travel expenses.

### Data analyses

#### Data reduction

In line with previous studies (e.g., [24]), RT’s < 200 ms and RT’s > 1000 ms were considered anticipatory responding and delayed responding, respectively, and were discarded. Non-response was considered a missing value and was discarded. An incorrect response was also discarded. Trials with less than 10 reaction times were discarded. Trial types (e.g., a positive valid trial with 500 ms presentation time) with 40% errors or more were also excluded. Statistical analyses were run on 96.24% of the data. Consistent with a series of recent studies using RT-based performance measures (e.g., [50, 51, 52]), we decided to use median instead of mean reaction times because this seems the most simple, straightforward, and robust way to deal with outliers without losing too much information. Median scores were computed for the different presentation times and type of trials (valid/invalid), for all stimulus types.

We computed separate indices of AB for each of the presentation times (500 and 1250 ms). The traditional AB scores were calculated using the formula suggested by Mogg et al.,[53]: Attentional bias score (AB score) = (median RT invalid emotional cue–median RT valid emotional cue)–(median RT invalid neutral cue–median RT valid neutral cue). At shorter presentation times of the cues (100–300 ms), faster responding is generally found on validly cued trials compared to invalidly cued trials, a finding that is referred to as the “cue validity” or cue facilitation effect. At longer presentation times (500–3000 ms), the cue facilitation effect disappears and even reverses because attention to the location of a previously attended stimulus is inhibited in favor of new locations. This is known as the inhibition of return effect (IoR;[54]). In the current emotional modification of this paradigm, the emotional value of the cue is varied (i.e., emotional vs. neutral) which allows to investigate AB for disorder-relevant emotional information (with the responses to the neutral trials as the reference category and the comparison control participants as the reference group). Therefore, more positive AB scores (i.e., stronger cue validity effects) were indicative of a stronger attentional bias towards the emotional information. Less negative AB scores were indicative of a weaker inhibition of return effect (see [55]). Relatively strong cue validity effects and relatively weak IoR (and thus more positive or less negative AB scores) are interpreted as attention bias toward a particular stimulus type, whereas relatively weak cue validity effects and strong IoR (and thus less positive or more negative AB scores) are considered to reflect a bias away from particular stimuli. Given the stimulus onset asynchrony used in the current study (500 and 1250 ms), negative cue validity effects were to be expected.

We computed an AB score for negative, threat, and positive words per presentation time. We considered indices deviating more than 3 SDs from the mean of the group as outliers for all the groups. We replaced these outliers with the group mean for that index plus (or minus) 3 SDs.

We computed trial-level bias scores (TL-BS) based on the computational methodology of Zvielli et al. [30]to examine the temporal dynamics of AB. We matched each invalid trial with a subsequently presented valid trial (thus, in a single direction from the beginning to the end of the task), temporally as close as possible and no further than 9 trials away from each other, for each stimulus type. Paired trials that were more than 9 trials apart were discarded. We used this same method to match each valid trial to a subsequently presented invalid trial. As such, a given trial was included in maximally two pairs maximum. In this way, we computed time series of TL-BS per participant. The number of trial types among which pairings have to be made is increased considerably from 3 in the dot probe task to 8 in the currently analyzed ECT. In line with the approach of Zvielli et al. [30], we calculated 5 indices of TL-BS based on the derived pairings for each stimulus type per 2 presentation times (40 TL-BS indices in total) which indicated individual differences in phasic bursts or “peaks” of AB expression, mean levels of TL-BS toward and away from target stimuli, and degree of TL-BS variability over time across the spectrum of AB (away, towards, or both). Per presentation time and per stimulus type (e.g., positive), we calculated for each participant (i) the mean TL-BS Towards (the mean of the TL-BS scores that were higher than 0 ms indicating attention towards the stimuli [i.e., invalid trial RT was higher than valid trial RT]), (ii) mean TL-BS Away (the mean of the TL-BS scores that were lower than 0 indicating attention away from the stimuli [i.e., valid trial RT was higher than invalid trial RT]), (iii) the peak TL-BS Towards (maximum TL-BS indicating an AB toward target stimuli [i.e., invalid trial RT was higher than valid trial RT]), (iv) peak TL-BS Away (minimum TL-BS indicating attention away from target stimuli [i.e., valid trial RT was higher than invalid trial RT]), and (v) variability (reflects the degree of stability or temporal variability in the expression of attention toward and/or away over time, calculated by the standard deviation of TL-BS). Within the current ECT there were differential lags between the various TL-BSs rendering it problematic to calculate TL-BS Variability in exactly the same way as Zvielli et al. [30] did (sum of all distances between sequential TL-BSs divided by the number of TL-BSs). To stay as close to the concept of TL-BS Variability as possible, we eventually decided to use the standard deviation of TL-BSs to index variability in TL-BS. Because indices were positively skewed, variables were subjected to a square root transformation, before being used in the analyses.

### Statistical analyses

To test the predicted pattern of stimulus specific AB as a function of group we subjected the traditional AB scores (Negative 500 ms, Negative 1250 ms, Positive 500 ms, Positive 1250 ms, Threat 500 ms, Threat 1250 ms) to a Multivariate Analyses of Variance (MANOVA) with the AB scores as the dependent factor and Group (Comparison, rMDD, MDD, and MDD/AD) as fixed factor. In a complementary approach, to test the temporal dynamics of the stimulus specificity of AB between the groups for the different stimuli types, we subjected (absolute) mean TL-BS scores and (absolute) peak TL-BS scores, and TL-BS Variability to similar MANOVA’s with TL-BS indices as dependent factor and Group (Comparison, rMDD, MDD, and MDD/AD) as fixed factor.

## Results

### Descriptives

For characteristics of participants as a function of group see Table 1. The groups neither differed significantly on age (*F* (3,167.54) = .24, *p* = .86), nor on gender, Pearson *χ*^2^ (3) = 6.74, *p* = .08.

The mean of the median reaction times per stimulus type and the cue validity effects are displayed in Table 2 as a function of trial type (valid vs. invalid; short vs. long duration) and group. For both short and long presentation times, participants were generally faster on invalid than on valid trials. The relatively slow reaction times on validly cued trials indicate an inhibition of return effect.

**Table 2:** Reaction times of participants per group per stimulus type per presentation time and cue validity effect (means and standard deviations) in milliseconds.

Group	Comparison			MDD			Mixed MDD/AD			rMDD		
	Invalid	Valid	CV	Invalid	Valid	CV	Invalid	Valid	CV	Invalid	Valid	CV
Stimuli												
500 ms												
Negative	376 (71)	401 (70)	-26 (48)	392 (69)	442 (99)	-50 (60)	392 (81)	423 (84)	-31 (56)	389 (67)	412 (74)	-22 (46)
Positive	367 (71)	406 (75)	-39 (49)	397 (81)	436 (93)	-38 (57)	394 (83)	428 (81))	-37 (43)	382 (69)	418 (76)	-36 (50)
Threat	367 (69)	405 (73)	-38 (47)	390 (70)	433 (88)	-43 (50)	397 (81)	425 (82)	-29 (51)	381 (69)	417 (76)	-36 (46)
Neutral	375 (71)	401 (77)	-25 (52)	406 (72)	434 (103)	-29 (62)	394 (84)	422 (90)	-27 (46)	390 (69)	414 (79)	-24 (52)
1250 ms												
Negative	390 (67)	433 (72)	-42 (43)	398 (58)	453 (71)	-55 (37)	407 (72)	457 (82)	-49 (48)	404 (69)	444 (75)	-39 (41)
Positive	391 (68)	429 (73)	-38 (41)	397 (56)	445 (73)	-47 (44)	405 (75)	453 (83)	-48 (48)	405 (70)	442 (76)	-36 (42)
Threat	397 (67)	442 (73)	-46 (42)	400 (62)	451 (74)	-49 (35)	412 (74)	465 (83)	-52 (51)	409 (72)	457 (78)	-48 (45)
Neutral	401 (71)	430 (75)	-28 (46)	404 (69)	443 (79)	-38 (43)	409 (74)	456 (81)	-46 (47)	411 (71)	445 (81)	-33 (44)

Note:

MDD = group of participants with Major Depressive Disorder

Mixed MDD/AD = group of participants with Major Depressive Disorder and Anxiety Disorder

rMDD = group of participants who are remitted from Major Depressive Disorder

CV = cue validity effect

### Traditional AB score

Table 3 gives a detailed description of the traditional AB score per stimulus type and presentation time.

**Table 3:** Traditional AB scores per group per stimulus type and per presentation time (means and standard deviations) in milliseconds.

GroupStimuli	Comparison	MDD	Mixed MDD/AD	rMDD
500 ms				
Negative	-1.08 (42.44)	-16.86 (57.79)	-1.56 (58.16)	1.79 (41.39)
Positive	-14.96 (44.23)	-2.75 (56.92)	-11.30 (45.24)	-11.89 (43.95)
Threat	-13.65 (40.62)	-11.90 (42.29)	-2.24 (54.94)	-12.20 (42.45)
1250 ms				
Negative	-14.61 (44.77)	-17.51 (42.77)	-3.14 (48.15)	-5.54 (44.67)
Positive	-9.71 (41.59)	-8.98 (45.55)	-1.93 (57.59)	-3.39 (47.39)
Threat	-17.75 (46.44)	- 11.35 (37.52)	-5.45 (45.53)	-14.37 (48.39)

Note:

MDD = group of participants with Major Depressive Disorder

Mixed MDD/AD = group of participants with Major Depressive Disorder and Anxiety Disorder

rMDD = group of participants who are remitted from Major Depressive Disorder

Table 4 shows the significant post hoc contrasts for the analyses of the traditional AB scores as well as of the TL-BS indices. The MANOVA showed a significant intercept (Wilks’ *λ* = .95, *F* (6, 848) = 6.70, *p* < .001, partial *η*^2^ = .04) indicating that overall the AB scores differed from zero. Thus supporting its validity, the ECT was sufficiently sensitive to detect differences in participants’ AB for neutral versus disorder-relevant stimuli. Most important for the current context, there was a significant multivariate effect of group (Wilks’ *λ* = .95, *F* (18, 2398.99) = 2.09, *p* < .004, partial *η*^2^ = .15. The between subject tests indicated that the multivariate effect of group was mainly carried by the AB index of negative adjectives specifically for long duration trials (1250 ms). Only for this AB index, the effect of group was significant (*F* (3,853) = 3.51, *p* = .01, partial *η*^2^ = .01). There was a non-significant trend for AB Threat 500 ms (*F* (3, 853) = 2.32, *p* = .07, partial *η*^2^ = .008). For all other AB scores there was no statistically significant between group difference; AB Negative 500 ms (*F* (3, 853) = 1.66, *p* = .17, partial *η*^2^ = .006), AB Positive 500 ms (*F* (3,853) = 0.86, *p* = .45, partial *η*^2^ = .003), AB Positive 1250 ms (*F* (3, 853) = 0.99, *p* = .39, partial *η*^2^ = .003), Threat 1250 ms (*F* (3, 853) = 1.63, *p* = .18, partial *η*^2^ = .006).

**Table 4:** Significant between group contrasts for traditional AB scores and TL-BS parameters.

Index AB	Groups	Mean difference	*p*	CIUnder bound	CIUpper bound	*d*
		Traditional AB				
Negative 1250 ms	rMDD- Comparison	9.07	.04	0.20	17.93	0.20
		TL-BS indices Mean				
Positive 1250 msAway	Mixed MDD/AD—Comparison	0.97	< .01	0.29	1.65	0.31
Threat500 msTowards	Mixed MDD/AD–Comparison	1.05	< .01	0.26	1.83	0.36
Threat 1250 ms Away	Mixed MDD/AD–Comparison	0.71	< .01	0.16	1.25	0.36
		TL-BS indices Variability				
Positive 500 ms	Mixed MDD/AD–Comparison	0.76	< .01	0.12	1.40	0.36
Positive 1250 ms	Mixed MDD/AD–Comparison	0.87	< .001	0.26	1.48	0.44
Neutral 1250 ms	Mixed MDD/AD–Comparison	-0.69	.02	-1.31	-0.06	0.32
Threat 500 ms	Mixed MDD/AD–Comparison	0.88	< .002	0.27	1.49	0.38
Threat 1250 ms	Mixed MDD/AD–Comparison	0.75	< .002	0.23	1.27	0.40
	rMDD- Comparison	0.35	.03	0.02	0.68	0.18

Note:

MDD = group of participants with Major Depressive Disorder

Mixed MDD/AD = group of participants with Major Depressive Disorder and Anxiety Disorder

rMDD = group of participants who are remitted from Major Depressive Disorder

Bonferrroni adjustedpost hoc between group tests indicated that for the 1250 ms trials specifically the rMDD group showed a higher (less negative) AB score for negative adjectives than the comparison group without (a history of) MDD/AD (mean difference 9.07 ms (s.e. 3.35), *p* = .04, 95% CI [0.20, 17.93], *d* = 0.20) (see also Table 4 for all significant between group contrasts). All other between group contrasts for AB Negative 1250 ms did not reach significance.

To examine whether in line with predictions, the effect of group for the Threat AB score Presentation Time 500 ms that just fell short of the conventional level of significance was mainly due to more extreme AB for threat in the mixed MDD/AD group, we used Dunnett’s method (two sided) for multiple comparisons. This involved a comparison of the AB index between each of the (sub)clinical groups and the comparison group [56]. In line with our hypothesis only the mixed MDD/AD group tended to differ from the comparison group (mean difference 11.41 ms (s.e. 5.13), *p* = .07, 95% CI [-0.84; 23.67], *d* = 0.23); indicating that the mixed MDD/AD group tended to show higher (less negative) AB-threat scores than the comparison group (see also Table 4). Neither the MDD group (mean difference 1.74 ms (s.e. 8.48), *p* = .99, 95% CI [-18.51; 21.99], *d* = 0.04) nor the rMDD group (mean difference 1.44 ms (s.e. 3.20), *p* = .955, 95% CI [-6.21; 9.10], *d* = 0.03), differed significantly from the comparison group with regard to the AB treat index.

### Temporal dynamics

For the correct trials and missing pairs in calculating TL-BS per stimulus type per presentation time, see S3 Appendix. For the TL-BS indices within each condition (Group, Index, Stimulus type, Presentation Time), see S4 Appendix.

To condense the result section, we restricted the report of the main analyses of the TL-BS to Mean TL-BS Towards and Mean TL-BS Away, and left out the analyses of the TL-BS peak indices, as there was a high correlation between Mean and Peak TL-BS parameters (*r* = .82 to .88); in line with this, Peak TL-BS Towards and Away indices showed the same pattern of results as those reported for Mean Towards and Away indices, respectively. See Table 5 for zero-order correlations.

**Table 5:** Zero-order correlations for traditional AB score, TL-BS parameters per presentation time.

IndexStimuli	Mean Toward	Mean Away	Peak Toward	Peak Away	Variability
		Presentation time 500 ms			
Negative					
Traditional AB	.08 **	-.07**	.11**	-.05*	-.02
Mean Toward		.20**	.87**	.15**	.66**
Mean Away			.10**	.85**	.72**
Peak Toward				.06**	.63**
Peak Away					.72**
Positive					
Traditional AB	.01	-.14**	.05*	-.12**	-.07**
Mean Toward		.17**	.88**	.11**	.63**
Mean Away			.07**	.84**	.73**
Peak Toward				.02	.59**
Peak Away					.72**
Threat					
Traditional AB	.11**	-.15**	.15**	-.14**	-.005
Mean Toward		.17**	.88**	.13**	.63**
Mean Away			.06**	.87**	.70**
Peak Toward				.01	.61**
Peak Away					.70**
Neutral					
Mean Toward		.21**	.87**	.12**	.68**
Mean Away			.12**	.84**	.73**
Peak Toward				.04	.64**
Peak Away					.68**
		Presentation time 1250 ms			
Negative					
Traditional AB	.06**	-.15**	.10**	-.15**	-.03
Mean Toward		.26**	.86**	.19**	.70**
Mean Away			.17**	.82**	.75**
Peak Toward				.09**	.67**
Peak Away					.71**
Positive					
Traditional AB	.07**	-.14**	.12**	-.15**	-.02
Mean Toward		.22**	.86**	.17**	.68**
Mean Away			.14**	.84**	.73**
Peak Toward				.06**	.65**
Peak Away					.72**
Threat					
Traditional AB	.05*	-.18**	.10**	-.14**	-.02
Mean Toward		.21**	.88**	.15**	.66**
Mean Away			.12**	.83**	.73**
Peak Toward				.05*	.63**
Peak Away					.70**
Neutral					
Mean Toward		.18**	.88**	.12**	.67**
Mean Away			.10**	.83**	.71**
Peak Toward				.03	.64**
Peak Away					.69**

*p< .05

** *p* < .01

#### TL-BS mean towards and away

Table 6 gives a detailed description of the TL-BS indices Mean and Variability scores per stimulus type and presentation time.

**Table 6:** TL-BS scores per group per stimulus type and per presentation time (means and standard deviations).

GroupTL-BS index	Comparisons	MDD	Mixed MDD/AD	Remitted
500 ms				
Negative				
Mean Towards	8.10 (2.37)	8.60 (3.09)	8.86 (2.68)	8.14 (2.32)
Mean Away	9.33 (2.32)	9.80 (2.50)	9.85 (2.17)	9.52 (2.36)
Variability	9.75 (2.08)	10.38 (2.12)	10.30 (2.04)	10.00 (2.09)
Positive				
Mean Towards	7.92 (2.39)	8.10 (2.45)	8.56 (2.57)	8.33 (2.40)
Mean Away	9.68 (2.34)	10.03 (2.42)	10.39 (2.22)	9.84 (2.32)
Variability	9.94 (2.09)	10.23 (1.78)	10.71 (2.13)	10.20 (2.00)
Threat				
Mean Towards	7.74 (2.63)	7.52 (3.22)	8.79 (3.16)	7.93 (2.65)
Mean Away	9.31 (2.38)	9.67 (2.73)	9.65 (2.68)	9.53 (2.45)
Variability	9.43 (2.08)	9.62 (2.20)	10.32 (2.54)	9.72 (2.21)
Neutral				
Mean Towards	8.40 (2.35)	9.16 (1.70)	8.66 (2.75)	8.47 (2.29)
Mean Away	9.44 (2.25)	9.84 (2.88)	9.88 (2.76)	9.47 (2.40)
Variability	10.09 (2.00)	10.56 (1.98)	10.71 (2.26)	10.10 (2.16)
1250 ms				
Negative				
Mean Towards	8.18 (2.34)	8.16 (2.02)	8.33 (2.37)	8.44 (2.41)
Mean Away	9.93 (1.91)	9.93 (2.12)	10.20 (2.17)	9.93 (2.07)
Variability	10.14 (1.95)	10.10 (2.005)	10.43 (1.95)	10.32 (1.98)
Positive				
Mean Towards	8.14 (2.43)	8.34 (2.33)	8.82 (2.53)	8.64 (2.61)
Mean Away	9.59 (2.15)	10.21 (2.40)	10.57 (2.34)	9.59 (2.20)
Variability	10.02 (1.91)	10.26 (1.91)	10.90 (2.03)	10.28 (2.04)
Threat				
Mean Towards	7.93 (2.46)	8.08 (2.53)	8.47 (2.75)	8.25 (2.45)
Mean Away	10.06 (1.91)	10.66 (2.11)	10.77 (2.01)	10.27 (1.99)
Variability	10.04 (1.85)	10.53 (2.17)	10.79 (1.81)	10.39 (1.85)
Neutral				
Mean Towards	8.22 (2.48)	8.71 (2.79)	8.87 (3.52)	8.70 (2.27)
Mean Away	9.62 (2.26)	10.21 (2.48)	10.18 (2.25)	9.70 (2.13)
Variability	9.98 (2.02)	10.54 (1.82)	10.68 (2.30)	10.32 (1.91)

Note:

MDD = group of participants with Major Depressive Disorder

Mixed MDD/AD = group of participants with Major Depressive Disorder and Anxiety Disorder

rMDD = group of participants who are remitted from Major Depressive Disorder

The MANOVA showed a significant multivariate effect of group (Wilks’ *λ* = .88, *F* (48, 2139) = 1.83, *p* < .001, partial *η*^2^ = .03). The between subject tests indicated that for the negative adjectives none of the TL-BS scores showed a significant difference between groups (Negative Away 500 ms *F* (3,734) = 1.20, *p* = .30, partial *η*^2^ = .005; Negative Towards 1250 ms *F* (3,734) = 1.68, *p* = .16, partial *η*^2^ = .007, and Negative Away 1250 ms *F* (3,734) = 1.15, *p* = .32, partial *η*^2^ = .005), though for Negative Towards 500 ms this just fell short of the conventional level of significance (Negative Towards 500 ms *F* (3,734) = 2.38, *p* = .06, partial *η*^2^ = .01). Post hoc Bonferroni adjusted between group tests indicated that none of the between group differences with regard to this AB index reached significance.

For the positive adjectives the between subject tests indicated a significant difference between groups for Positive Towards 500 ms *F* (3,734) = 3.74, *p* = .01, partial *η*^2^ = .01; Positive Towards 1250 ms *F* (3,734) = 3.04, *p* = .02, partial *η*^2^ = .01, and for Positive Away 1250 ms *F* (3,734) = 6.21, *p* < .001, partial *η*^2^ = .02. The between group effect for the TL-BS Positive Away 500 ms just fell short of significance *F* (3,734) = 2.40, *p* = .06, partial *η*^2^ = .01. Bonferrroni controlled post hoc between group contrasts indicated that for TL-BS Mean Positive 1250 ms Away the mean difference between the mixed MDD/AD group and the comparison group was 0.97 (s.e. 0.25), *p* < .01, 95% CI [0.29, 1.65], *d* = 0.31. All other mean differences between groups were not statistically significant. This pattern indicates that for presentation times 1250 ms the mixed MDD/AD group showed more extreme scores on TL-BS away from positive adjectives than the comparison group.

For the neutral words there was an unexpected yet significant difference between groups regarding TL-BS Neutral Towards 1250 ms, *F* (3,734) = 4.58, *p* = .003, partial *η*^2^ = .01. For the other indices regarding the neutral stimuli there were no significant between group differences (Neutral Towards 500 ms *F* (3,734) = 1.27, *p* = .28, partial *η*^2^ = .005; Neutral Away 500 ms *F* (3,734) = 0.47, *p* = .69, partial *η*^2^ = .002; and Neutral Away 1250 ms *F* (3,734) = 1.17, *p* = .31, partial *η*^2^ = .005). Bonferrroni adjusted post hoc tests tests indicated that none of the between group contrasts were statistically significant.

For threat words there were significant differences between groups for Threat Towards 500 ms *F* (3,734) = 4.80, *p* = .003, partial *η*^2^ = .01 and for Threat Away 1250 ms *F* (3,734) = 2.68, *p* = .04 partial *η*^2^ = .01. For the other indices of threat stimuli there were no significant between group differences (Threat Away 500 ms *F* (3,734) = .62, *p* = .60 partial *η*^2^ = .003; Threat Towards 1250 ms *F* (3,734) = 1.89, *p* = .13, partial *η*^2^ = .008). To examine whether in line with predictions, the effect of group was mainly due to more extreme AB for threat in the mixed MDD/AD group we used Dunnett’s method for multiple comparisons (two sided). For TL-BS Mean Threat 500 ms Towards, the mixed MDD/AD group showed significantly higher scores than the comparison group with a mean difference of 1.05 (s.e. 0.32), *p* < .01, 95% CI [0.26; 1.83], *d* = 0.36. Neither the MDD group (mean difference = -0.22 (s.e. 0.52), *p* = .96, 95% CI [-1.47; 1.01], *d* = 0.07) nor the rMDD group (mean difference 0.18 (s.e. 0.20), *p* = .75, 95% CI [-0.31; 0.68], *d* = 0.07) showed significantly higher scores than the comparison group. For TL-BS Mean Threat 1250 ms Away we found the same pattern; the mixed MDD/AD group showed higher scores than the comparison group (mean difference 0.71 (s.e. 0.22), *p* = .006, 95% CI [0.16; 1.25], *d* = 0.36). For neither the MDD group (mean difference 0.59 (s.e. 0.37), *p* = .29, 95% CI [-.29; 1.49], *d* = 0.29) nor the rMDD group (mean difference 0.20 (s.e. 0.14), *p* = .37, 95% CI [-.13; 0.55], *d* = 0.10), the TL-BS Mean Threat 1250 ms Away scores differd from those of the comparison group. Together, this pattern indicates that the mixed MDD/AD group showed larger AB scores towards shortly presented threat words as well as larger AB scores away from longer presented threat words than the comparison group.

#### TL-BS variability

The MANOVA showed a significant multivariate effect of group (Wilks’ *λ* = .95, *F* (24, 2506) = 1.82, *p* < .01, partial *η*^2^ = .01. The between subject tests indicated that the effect of group was neither significant for variability of AB for Negative 500 ms (*F* (3,871) = 2.61, *p* = .05, partial *η*^2^ = .009), nor for Negative 1250 ms (*F* (3,871) = .86, *p* = .45, partial *η*^2^ = .003). For the positive adjectives there was a significant difference between groups for Positive 500 ms (*F* (3,871) = 3.74, *p* = .01, partial *η*^2^ = .01) and for Positive 1250 ms (*F* (3,871) = 5.63, *p* = .001, partial *η*^2^ = .01). Bonferroni adjusted post hoc between group tests indicated that for TL-BS Variability Positive 500 ms the comparison group and the mixed MDD/AD group differed significantly with a mean difference of 0.76 (s.e. 0.24), *p* < .01, 95% CI [0.12, 1.40], *d* = 0.36. For TL-BS Variability Positive 1250 ms, the mean difference between the comparison group and the mixed MDD/AD group was 0.87 (s.e. 0.23), *p* < .001, 95% CI [0.26, 1.48], *d* = 0.44 (see also Table 4). This pattern indicates that for both presentation times the mixed MDD/AD group showed more variability on positive word trials than the comparison group.

For the neutral words there was an unexpected yet significant difference between groups for Neutral 1250 ms (*F* (3,871) = 4.11, *p* = .007, partial *η*^2^ = .01). The difference between groups for Neutral 500 ms just fell short of significance (*F* (3,871) = 2.53, *p* = .05, partial *η*^2^ = .009). Bonferroni adjusted post hoc tests indicated that for TL-BS Variability Neutral 500 ms none of the groups differences were significant. For TL-BS Variability Neutral 1250 ms the mean difference between the comparison group and the mixed MDD/AD group was -0.69 (s.e. 0.23), *p* = .02, 95% CI [-1.31, -0.06], *d* = 0.32. This pattern indicates that for the long presentation time (1250 ms) the mixed MDD/AD group showed less variability than the comparison group on neutral word trials.

For Threat 500 ms *F* (3,871) = 5.16, *p* = .002, partial *η*^2^ = .01 and for Threat 1250 ms *F* (3,871) = 4.85, *p* = .002, partial *η*^2^ = .009 there was a significant difference between groups. To examine whether in line with predictions, the effect of group was mainly due to more variability for threat in the mixed MDD/AD group we used Dunnett’s method for multiple comparisons (two sided). For TL-BS Variability Threat 500 ms the mixed MDD/AD group showed significantly more variability than the comparison group with a mean difference of 0.88 (s.e. 0.25), *p* < .01, 95% CI [0.27; 1.49], *d* = 0.38. Neither the MDD group (mean difference 0.18 (s.e. 0.41), *p* = .95, 95% CI [-0.81; 1.18], *d* = 0.08) nor the rMDD group (mean difference 0.28 (s.e. 0.16), *p* = .21, 95% CI [-0.27; 1.49], *d* = 0.13) showed significantly stronger variability than the comparison group. Also for TL-BS Variability Threat 1250 ms the mixed MDD/AD group showed significantly more variability than the comparison group with a mean difference of 0.75 (s.e. 0.21), *p* < .001, 95% CI [0.23; 1.27], *d* = 0.40. Unexpectedly, also the rMDD group showed more variability than the comparison group with a mean difference of 0.35 (s.e. 0.13), *p* < .03, 95% CI [0.02; 0.68], *d* = 0.18 (see Table 4). The MDD group (mean difference 0.48 (s.e. 0.35), *p* = .42, 95% CI [-0.36; 1.34], *d* = 0.24) did not differ significantly from the comparison group. This pattern indicates that for both presentation times the mixed MDD/AD group showed more variability in AB for general threat words than the comparison group, whereas the rMDD group also showed more variability in AB for general threat words than the comparison group but only for short duration trials (500 ms).

## Discussion

This study investigated depression-related AB within the context of a large scale nationwide study on depression and anxiety, which allowed us to select rigorously-defined clinical groups. These groups consisted of participants with pure MDD without a history of AD, participants with both MDD and AD (mixed group), and individuals who were remitted from MDD (rMDD). These clinical groups were contrasted with participants without a history of MDD or ADs. The main results using traditional AB scores were: (i) compared to those without a history of MDD or AD, there was no evidence for a difference in AB towards negative adjectives or away from positive adjectives in strictly defined clinical groups of MDD participants with or without a comorbid AD; (ii) specifically for longer duration trials (1250 ms), rMDD individuals showed higher (less negative) AB scores for negative adjectives than the no AD/MDD comparison group. Also when indexed by trial-level bias scores (iii) there was no evidence for a relatively strong AB for negative adjectives in participants with MDD or mixed MDD/AD; (iv) specifically the mixed MDD/AD group showed higher and more variable mean AB scores towards shortly (500 ms) and away from longer (1250 ms) presented general threat words than the no MDD/AD comparison group; (v) The mixed MDD/AD group showed more variability on positive word trials, and specifically for the longer presentation trials (1250 ms) also higher scores for the AB index away from positive adjectives than the comparison group. Below we discuss these findings in relation to the key issues that this study aimed to address.

### Stimulus specificity of AB in MDD

This study tested the presence of AB for negative adjectives in MDD. Since a negative self-concept constitutes a critical component of cognitive models of depression (e.g., [7], AB for negative adjectives could be one of the mechanisms involved in the persistence of MDD. Against predictions, there was no specific AB in the group of MDD, neither for negative nor for positive adjectives. This was true for both AB quantified by traditional AB indices, and AB quantified by trial-level bias scores (TL-BS). Thus, the current results for a well-defined clinical group of participants with MDD selected from a large multi-center sample did not corroborate previous research using a similar ECT in high versus low dysphoric students [24]. The current study also failed to corroborate the findings of an earlier small-scale visual probe study (using 500 ms presentation time) among individuals with dysthymia (n = 13) or MDD (n = 7) indicating that these participants were characterized by a vigilance for adjectives that were very similar to the ones used in the current study (e.g., inadequate, useless, stupid, inept) [19]. Similarly, the current results also seem at odds with previous work in clinical groups of participants with MDD which did show an attentional bias for negative stimuli as indexed by sad and angry faces [29, 44]. The current findings cast some doubt on the robustness of these earlier findings. One explanation could be that the current MDD group consisted of people without a comorbid (or history of) AD, whereas in the previous research, selection of participants was less stringent. However, this does not seem to be a very convincing explanation, since the mixed group that was included in the current design did not show an AB for negative adjectives either. Perhaps, then, individuals with MDD are especially prone to direct their attention towards negative and/or depression-related interpersonal signals (e.g., facial expressions), but not so much to stimuli that are more specifically related to a negative self-concept per se. One way to test this explanation would be to use both types of stimuli within a single study.

### AB in participants with mixed depressive disorder and anxiety disorder(s)

None of the analyses using the traditional AB indices showed a specific AB in the mixed group, neither for negative nor for positive stimuli. Yet, the current findings did provide evidence for the predicted AB for general threat words in the mixed group of MDD with AD(s) as indexed by trial-level AB scores, whereas for the traditional AB index, the specific contrast between the mixed MDD/ADD group and the comparison group only showed a non-significant tendency suggesting that specifically for the short duration trials (500 ms) the mixed group showed a heightened (less negative) bias score. For the short duration trials, the TL-BS findings indicated that specifically the mixed MDD/AD group showed heightened scores for the index of mean AB towards threat stimuli. For longer duration trials, specifically the mixed MDD/AD group showed heightened scores for the index of mean AB away from threat stimuli. This pattern of findings with regard to the threat stimuli is consistent with a vigilance-avoidance pattern that has been previously reported in the context of threat scenes (e.g., [57]). In addition, for both presentation times the mixed MDD/AD group showed more variability in AB scores for general threat words than the comparison group which may be interpreted as further evidence for a heightened sensitivity for threat stimuli (cf. [31]). Together the current pattern of findings regarding the threat trials is consistent with -and complements- previous studies showing that participants with AD are characterized by an AB for disorder-specific threat stimuli [53]. The heightened AB scores for threat stimuli in the mixed group could well be the result of the comorbid AD (e.g., driven by AD-related fearful preoccupations), although it cannot be ruled out that the presence of the threat bias is related to the more severe condition of the mixed group compared to the pure MDD group. The latter explanation would also be consistent with the finding that specifically participants of the mixed MDD/AD group also showed heightened TL-BS scores away from positive stimuli. The threat bias in the mixed group could also be a premorbid characteristic, one that might have contributed to the development of the anxiety symptoms in this group via enhancing anxiety vulnerability (cf. [58, 59]). It is important for future research to use a longitudinal approach to test whether indeed AB for general threat cues has predictive validity for the development of ADs.

### AB in rMDD participants

Interestingly, the rMDD group showed reduced inhibition of return for negative adjectives (and thus less bias away from negative adjectives) than the comparison group as reflected in higher (less negative) traditional AB scores than the comparison group. To the extent that one is willing to see less IoR (and thus less bias away from negative stimuli) as a stronger inclination to dwell on negative stimuli (cf. [55]), this AB may reflect a heightened sensitivity for negative adjectives. One explanation for such heightened sensitivity in remitted individuals might be that these negative adjectives may be related to the impending threat of a relapse. Obviously, this explanation remains speculative at this stage, and it would require testing whether such AB is indeed related to individuals’ concerns about a recurrent episode when exposed to this type of negative adjectives. Irrespective of its source, it seems plausible to assume that heightened attention for negative adjectives might promote the generation of a negative self-view, thereby lowering the threshold for the recurrence of depression, as described in the cognitive model of Beck [6, 7]. An important next step would be to use a longitudinal design to test whether indeed heightened AB in rMDD individuals is predictive of relapse. Unexpectedly, the group of remitted participants also showed evidence for a threat bias as indexed by heightened variability in AB for general threat words (1250 ms trials). One way to test further the relevance of this finding would be to examine if AB for general threat stimuli may heighten the chance of recurrence and might heighten the probability of the development of (comorbid) anxiety disorders. If AB for negative adjectives and/or general threat stimuli would set people at risk for recurrence, this would provide an important lead for clinicians to better tailor their interventions to prevent recurrent episodes of both depressive and anxiety disorders.

### Traditional mean AB scores vs. trial-level AB scores

It has been theorized that TL-BS would be superior in capturing AB to the traditional AB scores. There has been empirical evidence supporting this assumption in recent studies[30, 31], and this was the reason why we calculated AB as a stable factor using the traditional AB score and as a temporal dynamic factor, using TL-BS. Recently, evidence on the basis of a simulation study pointed to potential problems of the current TL-BS approach. Most important, the findings of this simulation study indicated that TL-BS indices are prone to result in false positive group differences; the differences between groups might in fact reflect differences in mean reaction times and/ or differences in overall SD [33].

In accordance with the view that the traditional and the current temporal indices represent different aspects of AB, the correlations between these indices were very small. Although, the overall pattern as a function of group was quite similar for both types of indices there were also some notable exceptions: The AB for negative stimuli in rMDD that was only evident for the traditional index whereas the AB away from positive stimuli in the mixed group and the biases for threat stimuli were only evident for the TL-BS. The findings of the TL-BS should however be interpreted with care. Apart from the conceptual criticisms with regard to TL-BS (e.g.,[33]), it is important to note that we measured AB with an ECT instead of a visual probe task (VPT), and used four instead of three categories of stimuli (as[30, 31]). This has limited the amount of data points to express TL-BS and the number of different trial types tripled, which could both have influenced the reliability and validity of these indices in our study. Moreover, the traditional AB-index reflects differential responding to emotional versus neutral cue words, whereas the current TL-BS indices reflect differential responding to validly and invalidly cued trials for each of the cue word types separately. Thus, although our findings did not provide straightforward support for the usefulness of TL-BS as an index of the temporal dynamics in AB that might have superior power over traditional AB indices on negative adjectives to differentiate between groups, it cannot be seen as a critical challenge to the relevance of indices that take temporal dynamics into account.

### Limitations

Several limitations of this study need to be considered. First, we used the ECT to measure AB; just like VPT, this paradigm is not optimally suited to differentiate between enhanced engagement and difficulty to disengage. Because these processes might play a different role in MDD, it would be interesting in future research to use a task that is especially designed for this (e.g., the Attentional Response to Distal vs. Proximal Emotional Information) [60]. Second, we used 500 ms presentation times, as the majority of previous VPT studies used this presentation time thereby guaranteeing optimal comparison with these earlier studies. To test whether differences in AB would be most pronounced when stimuli would be presented for a longer duration as was found in previous analogue research (e.g., [24]), we also included trials with 1250 ms presentation time. It is possible that other results would have emerged if we had used shorter or longer presentation times. Third, this study focused on a task where adjectives were task-irrelevant. In this way, the ECT measured the tendency to automatically (non-intentionally) focus attention on stimuli; perhaps more controlled (overt) spontaneous AB processes that can be indexed in free viewing tasks (e.g.,[61]) are more important in MDD. Fourth, some of the observed effects had a small effect size and might not easily replicate in smaller samples and some of the groups were relatively small, which implied that for some between group contrasts (e.g., MDD vs. comparison) the statistical power was insufficient to reliably detect differences in AB with moderate or small effect sizes. It is noteworthy, though, that the current sample size is an improvement on many of the previous studies. We deliberately choose to select participants with MDD and without dysthymia, allowing to specifically test AB in MDD per se. This strategy meant that we had to exclude a high number of individuals (suffering from MDD and dysthymia) and that the severity of depressive symptoms was less in the current MDD group than in the comorbid group.

Furthermore, the total sample of participants with MDD was too small to reliably examine sex differences in AB. In addition, many factors apart from AB may contribute to the development of MDD. To the extent that these factors do not exert their influence via attentional bias, this may have reduced the sensitivity of the current study to find between group differences in AB. Fifth, we translated the TL-BS from a VPT using 3 categories to this ECT using 4 categories of stimuli. In the process of translating we had to make pragmatic choices which might have been suboptimal; more generally the current task was not optimally suited to examine the relevance of TL-BS indices. Finally, as this study used a cross sectional design, it is not possible to draw conclusions about the direction of the relationships that were evident in this study.

## Conclusions

This study found no consistent evidence for AB towards negative adjectives or away from positive adjectives in strictly defined clinical groups of MDD participants with or without a comorbid AD. Thus, heightened AB for negative or a lowered AB for positive adjectives seems not to be critically involved in the maintenance of MDD. There was converging evidence indicating that individuals with mixed MDD/AD showed an AB for general threat words that reflected a vigilance-avoidance pattern. This is consistent with the view that threat bias is a premorbid characteristic that heightens the risk for the development of ADs, although it may also reflect a symptom of AD or the relatively high severity of the mixed group’s condition. The current findings provide preliminary evidence indicating that individuals who were remitted from MDD show an AB for negative adjectives and seem more sensitive for general threat stimuli. Both biases may reflect a heightened sensitivity for signals related to the impending threat of a new upcoming depressive episode among those who are currently remitted from MDD. Such heightened sensitivity for negative stimuli might well lower the threshold for entering a negative spiral ending up in the recurrence of depression. It would be important for future research to test whether attentional biases for negative adjectives and/or general threat stimuli are predictive for the recurrence of depression.

## Supporting information

S1 Appendix ECT Stimulus words: 16 stimulus words per stimulus type. (DOCX)

S2 Appendix ECT: Example of a valid trial. (DOCX)

S3 Appendix Correct pairs, missing pairs and pairs per index in calculating TL-BS per stimulus type per presentation time. (DOCX)

S4 Appendix Temporal dynamic AB score per group per index per stimulus type per presentation time; means and standard deviations in milliseconds. (DOCX)
