# Supplementary material for: Attentional bias for negative, positive, and threat words in current and remitted depression
Source: PLoS One. 2018 Oct 31;13(10):e0205154. doi: 10.1371/journal.pone.0205154 (PMC6209165; doi:10.1371/journal.pone.0205154)
Supplement: S1 Appendix — (DOCX) [file pone.0205154.s001.docx]

S1 Appendix ECT Stimulus words: 16 stimulus words per stimulus type

| **Stimulus type** | **Woorden (Dutch)** | **Words (English)** |
| --- | --- | --- |
| Positive | Opgewekt  levenslustig  succesvol  positief  populair  krachtig  waardevol  blij  winnaar  vlot  actief  geliefd  optimistisch  energiek  zelfbewust  talentvol | cheerful  bright  successful  positive  popular  powerful  valued  happy  winner  outgoing  dynamic  beloved  optimistic  industrious  assertive  talented |
| Threat | terrorist  invalide  dodelijk  fataal  hulpeloos  bedreiging  vijandig  gevaarlijk  aanval  kritiek  hersentumor  afwijzing  kanker  ziekte  verraden  pijn | terrorist  invalid  lethal  fatal  helpless  threat  hostile  dangerous  attack  criticism  brain tumor  rejection  cancer  disease  betrayed  pain |
| Negative | Negatief  ongewenst  ellendig  wanhopig  leeg  saai  zinloos  afgewezen  nutteloos  pessimistisch  eenzaam  verloren  somber  minderwaardig ongeschikt  waardeloos | negative  unwanted  vile  desperate  hollow  dull  aimless  rejected  useless  pessimistic  lonely  lost  morbid  inferior  incompetent  worthless |
| Neutral | Kapstok  dynamo  paperclip  behang  kantoorgebouw  potlood  plank  papieren  kraan  woordenboek  spatiebalk  lichtknopje tandenborstel  braadpan  zonnebril  omgeving | coat rack  dynamo  paperclip  wallpaper  office  pencil  shelf  papers  tap/crane  dictionary  spacebar  light switch  toothbrush  casserole  sunglasses  surroundings |
