# Supplementary figures and images for: Attentional bias for negative, positive, and threat words in current and remitted depression
Source: PLoS One. 2018 Oct 31;13(10):e0205154. doi: 10.1371/journal.pone.0205154 (PMC6209165; doi:10.1371/journal.pone.0205154)

S2 Appendix ECT : Example of a valid trial


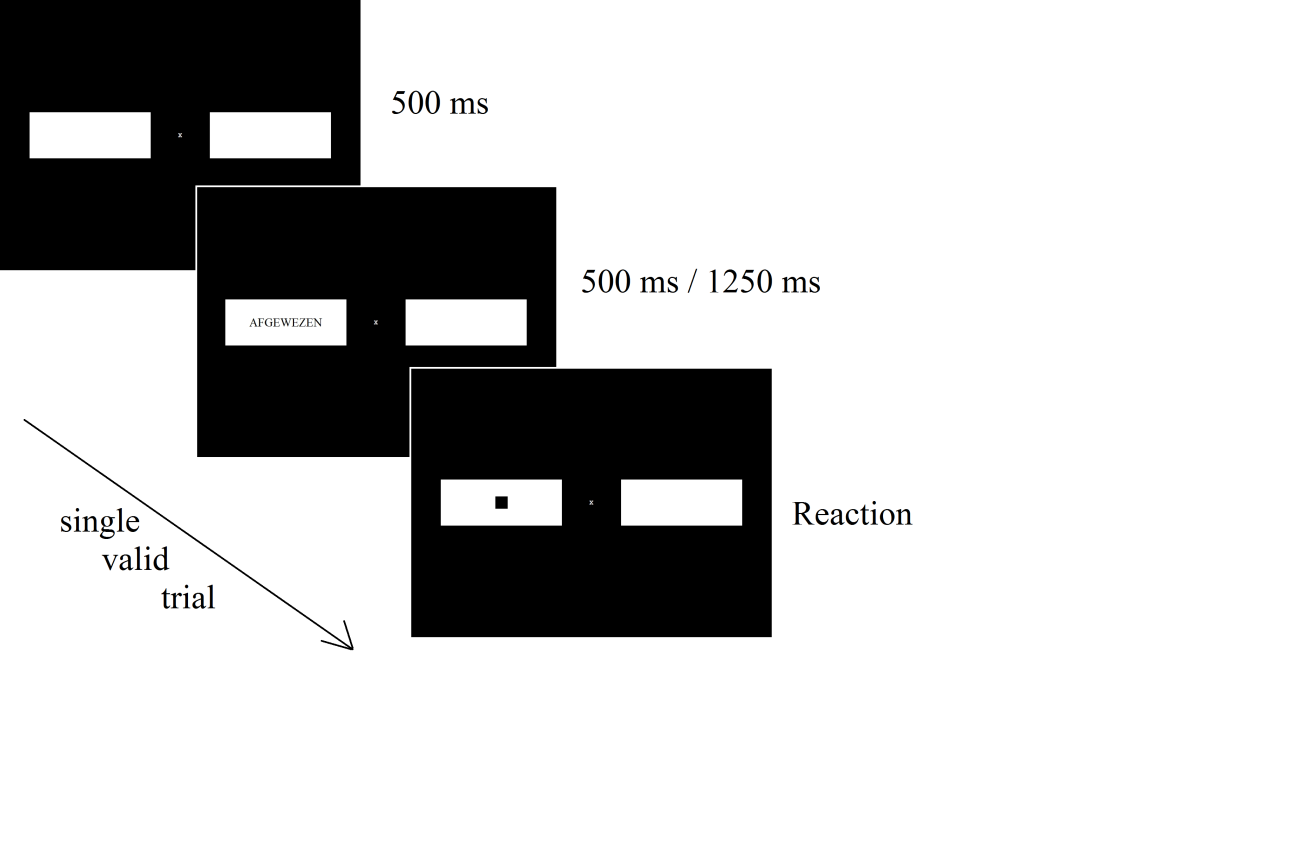

Supplement: S2 Appendix — (DOCX) [file pone.0205154.s002.docx]
