# Supplementary material for: Attentional bias for negative, positive, and threat words in current and remitted depression
Source: PLoS One. 2018 Oct 31;13(10):e0205154. doi: 10.1371/journal.pone.0205154 (PMC6209165; doi:10.1371/journal.pone.0205154)
Supplement: S3 Appendix — (DOCX) [file pone.0205154.s003.docx]

S3 Appendix. Correct Trials and Miss Pairs in Calculating TL-BS per Stimulus Type per Presentation Time

Presentation Time 500 ms

|  | N | Minimum | Maximum | Mean |
| --- | --- | --- | --- | --- |
| Stimulus Type |  |  |  |  |
| Correct Tails |  |  |  |  |
| Negative | 883 | .00 | 16.00 | 14.82 (2.13) |
| Positive | 883 | .00 | 16.00 | 14.77 (2.25) |
| Threat | 883 | .00 | 12.00 | 11.24 (1.52) |
| Neutral | 883 | .00 | 18.00 | 16.55 (2.45) |
|  |  |  |  |  |
| Miss Pairs |  |  |  |  |
| Negative | 883 | .00 | 16.00 | 1.17 (2.13) |
| Positive | 883 | .00 | 16.00 | 1.22 (2.25) |
| Threat | 883 | .00 | 12.00 | .75 (1.52) |
| Neutral | 883 | 1.00 | 19.00 | 2.44 (2.45) |
| Valid N (listwise) | 883 |  |  |  |

Presentation Time 1250 ms

|  | N | Minimum | Maximum | Mean |
| --- | --- | --- | --- | --- |
| Stimulus type |  |  |  |  |
| Correct Trails |  |  |  |  |
| Negative | 883 | .00 | 18.00 | 17.24 (1.75) |
| Positive | 883 | .00 | 12.00 | 11.50 (1.12) |
| Threat | 883 | .00 | 18.00 | 17.20 (1.68) |
| Neutral | 883 | .00 | 12.00 | 11.55 (1.08) |
|  |  |  |  |  |
| Miss Pairs |  |  |  |  |
| Negative | 883 | .00 | 18.00 | .75 (1.75) |
| Positive | 883 | .00 | 12.00 | .49 (1.12) |
| Threat | 883 | .00 | 18.00 | .79 (1.68) |
| Neutral | 883 | .00 | 12.00 | .44 (1.08) |
| Valid N (listwise) | 883 |  |  |  |
