# Supplementary material for: Attentional bias for negative, positive, and threat words in current and remitted depression
Source: PLoS One. 2018 Oct 31;13(10):e0205154. doi: 10.1371/journal.pone.0205154 (PMC6209165; doi:10.1371/journal.pone.0205154)
Supplement: S4 Appendix — (DOCX) [file pone.0205154.s004.docx]

S4 Appendix. Temporal Dynamic AB Score per Group per Index per Stimulus Type per Presentation Time; Means and Standard Deviations in milliseconds

Presentation Time 500 ms

| Group  Index TL-BS per Stimuli | Comparison | MDD | Mixed | rMDD |
| --- | --- | --- | --- | --- |
| Negative |  |  |  |  |
| Mean Towards | 8.10 (2.37) | 8.60 (3.09) | 8.20 (2.42) | 8.14 (2.32) |
| Mean Away | -9.33 (2.31) | -9.80 (2.50) | -9.46 (2.31) | -9.52 (2.33) |
| Peak Towards | 11.63 (3.76) | 12.26 (4.55) | 11.78 (3.93) | 11.08 (4.07) |
| Peak Away | -14.17 (3.98) | -14.59 (4.17) | -14.36 (3.96) | -14.56 (3.99) |
| Variance | 9.75 (2.08) | 10.38 (2.12) | 9.90 (2.09) | 10.00 (2.09) |
| Positive |  |  |  |  |
| Mean Towards | 7.92 (2.39) | 8.10 (2.45) | 8.56 (2.57) | 8.33 (2.40) |
| Mean Away | -9.67 (2.31) | -10.03 (2.42) | -10.39 (2.22) | -9.84 (2.32) |
| Peak Towards | 11.28 (4.01) | 11.93 (4.09) | 12.19 (3.92) | 11.88 (3.80) |
| Peak Away | -14.62 (3.86) | -14.61 (3.74) | -15.94 (3.67) | -14.96 (3.87) |
| Variance | 9.94 (2.09) | 10.23 (1.78) | 10.71 (2.13) | 10.20 (2.00) |
| Threat |  |  |  |  |
| Mean Towards | 7.74 (2.63) | 7.52 (3.22) | 8.79 (3.16) | 7.93 (2.65) |
| Mean Away | -9.30 (2.33) | -9.67 (2.73) | -9.65 (2.68) | -9.53 (2.45) |
| Peak Towards | 10.47 (4.00) | 9.80 (4.09) | 12.52 (5.05) | 10.79 (3.99) |
| Peak Away | -13.59 (3.77) | -14.03 (4.84) | -13.91 (3.88) | -13.83 (3.84) |
| Variance | 9.43 (2.08) | 9.62 (2.20) | 10.32 (2.54) | 9.72 (2.21) |
| Neutral |  |  |  |  |
| Mean Towards | 8.40 (2.35) | 9.16 (1.70) | 8.66 (2.75) | 8.47 (2.29) |
| Mean Away | -9.43 (2.21) | -9.84 (2.88) | -9.88 (2.76) | -9.47 (2.39) |
| Peak Towards | 12.40 (3.95) | 14.05 (4.42) | 12.91 (4.93) | 12.28 (3.77) |
| Peak Away | -14.56 (4.03) | -14.60 (4.11) | -15.02 (4.32) | -14.61 (4.26) |
| Variance | 10.09 (2.00) | 10.56 (1.98) | 10.71 (2.26) | 10.10 (2.16) |

Presentation Time 1250 ms

| Group  Index TL-BS per Stimuli | Comparison | MDD | Mixed | rMDD |
| --- | --- | --- | --- | --- |
| Negative |  |  |  |  |
| Mean Towards | 8.18 (2.34) | 8.16 (2.02) | 8.33 (2.37) | 8.44 (2.41) |
| Mean Away | -9.93 (1.90) | -9.93 (2.12) | -10.19 (2.13) | -9.91 (1.96) |
| Peak Towards | 11.88 (3.85) | 11.81 (3.12) | 12.23 (4.20) | 12.27 (4.05) |
| Peak Away | -15.42 (3.40) | -15.30 (3.78) | -15.69 (3.69) | -15.27 (3.49) |
| Variance | 10.14 (1.95) | 10.10 (2.00) | 10.43 (1.95) | 10.32 (1.98) |
| Positive |  |  |  |  |
| Mean Towards | 8.14 (2,43) | 8.34 (2.33) | 8.82 (2.53) | 8.64 (2.61) |
| Mean Away | -9.59 (2.14) | 12.17 (4.16) | -10.57 (2.34) | -9.60 (2.16) |
| Peak Towards | 11.24 (3.76) | 12.19 (4.26) | 12.14 (3.90) | 12.17 (4.16) |
| Peak Away | -14.18 (3.66) | -14.74 (3.94) | -15.47 (3.90) | -14.03 (3.47) |
| Variance | 10.02 (1.91) | 10.26 (1.91) | 10.90 (2.03) | 10.28 (2.04) |
| Threat |  |  |  |  |
| Mean Towards | 7.93 (2.46) | 8.34 (2.33) | 8.47 (2.75) | 8.25 (2.45) |
| Mean Away | -10.06 (1.91) | -10.21 (2.40) | -10.77 (2.01) | -10.28 (1.95) |
| Peak Towards | 11.24 (3.97) | 12.19 (4.26) | 11.89 (4.03) | 11.86 (3.98) |
| Peak Away | -15.59 (3.37) | -14.74 (3.94) | -16.67 (3.26) | -15.93 (3.41) |
| Variance | 10.04 (1.85) | 10.26 (1.91) | 10.79 (1.81) | 10.39 (1.85) |
| Neutral |  |  |  |  |
| Mean Towards | 8.22 (2.48) | 8.71 (2.79) | 8.87 (3.52) | 8.70 (2.27) |
| Mean Away | -9.62 (2.25) | -10.20 (2.45) | -10.18 (2.25) | -9.69 (2.11) |
| Peak Towards | 11.36 (3.96) | 12.42 (4.22) | 12.02 (4.91) | 12.14 (3.57) |
| Peak Away | -14.00 (3.50) | -14.69 (3.64) | -14.74 (3.70) | -14.11 (3.37) |
| Variance | 9.98 (2.02) | 10.54 (1.82) | 10.68 (2.30) | 10.32 (1.91) |
